# Peptides as Potential Therapeutics for Alzheimer’s Disease

**DOI:** 10.3390/molecules23020283

**Published:** 2018-01-30

**Authors:** Samo Ribarič

**Affiliations:** Institute of Pathophysiology, Faculty of Medicine, Zaloška 4, SI-1000 Ljubljana, Slovenia; samo.ribaric@mf.uni-lj.si; Tel.: +386-(0)1-543-7020

**Keywords:** aggregation, Alzheimer’s disease, amyloid β oligomers, amyloid β peptide, amyloid β plaques, insulin, neurofibrillary tangles, tau protein, peptides, peptide therapy

## Abstract

Intracellular synthesis, folding, trafficking and degradation of proteins are controlled and integrated by proteostasis. The frequency of protein misfolding disorders in the human population, e.g., in Alzheimer’s disease (AD), is increasing due to the aging population. AD treatment options are limited to symptomatic interventions that at best slow-down disease progression. The key biochemical change in AD is the excessive accumulation of per-se non-toxic and soluble amyloid peptides (Aβ(1-37/44), in the intracellular and extracellular space, that alters proteostasis and triggers Aβ modification (e.g., by reactive oxygen species (ROS)) into toxic intermediate, misfolded soluble Aβ peptides, Aβ dimers and Aβ oligomers. The toxic intermediate Aβ products aggregate into progressively less toxic and less soluble protofibrils, fibrils and senile plaques. This review focuses on peptides that inhibit toxic Aβ oligomerization, Aβ aggregation into fibrils, or stabilize Aβ peptides in non-toxic oligomers, and discusses their potential for AD treatment.

## 1. The Social and Economic Relevance of Alzheimer’s Disease

The increase in life expectancy is accompanied with an increased number of patients suffering from Alzheimer’s disease (AD). Patients with AD experience a progressive memory loss combined with a decline in cognitive function and ultimately a premature death 3–9 years after diagnosis [1]. AD is the most common cause of dementia, with a prevalence that increases with age to about 50% in people aged 85 or older. In practical terms, the majority of the population will have AD provided he or she has a sufficiently long life. The worldwide number of patients with AD is projected to increase to more than 140 million in 2050 [2].The total estimated worldwide cost of dementia in 2015 was US $818 billion, will reach US $1 trillion in 2018 and US $2 trillion by 2030. The true number of patients and the associated cost of dementia are likely to be considerably higher, since a huge majority of people with dementia have not received a diagnosis, have not been registered, and are thus unable to access care and treatment [3].

## 2. Etiology of AD

The characteristic changes in the brain tissue of AD patients are: (a) hyperphosphorylated tau protein rich intra-neuronal neurofibrillary tangles (NFT); (b) extracellular senile plaques of aggregated amyloid β (Aβ) peptides; and (c) brain tissue atrophy, starting in the entorhinal region and the temporal lobe, and progressing to the limbic system and the neocortex [4,5,6,7,8,9]. Most patients with AD have a sporadic, late onset form, where the major risk factors are aging, type 2 diabetes (T2D) and apolipoprotein E ε4 (APOE-ε4) [10,11,12,13,14,15]. A minority of AD patients have an early onset, genetic, familial form of AD due to autosomal dominant mutations in amyloid β precursor protein (AβPP), presenilin-1 (PS1), and presenilin-2 (PS2) [16]. The initiating step that leads to the disease in most AD patients is not known. The number and extent of amyloid plaques have only a week correlation with the degree of cognitive decline; they can also be found in individuals with mild cognitive impairment and in people with no cognitive symptoms [17]. There are at least three possible, and non-excluding, explanations for the lack of correlation between Aβ plaques and cognitive impairment in patients with AD: (a) person-to-person differences in the ability of inflammatory cells to effectively remove senile plaques from the brain [18]; (b) the high neurotoxic properties of Aβ42 oligomers, which precede the less neurotoxic senile plaques, are the main cause of cognitive impairment; and (c) person-to-person variations in brain plasticity, the ability to restore brain function after injury that operates over long [19] and short [20] timescales.

### 2.1. Amyloid Precursor Protein (APP)

**Figure 1:**
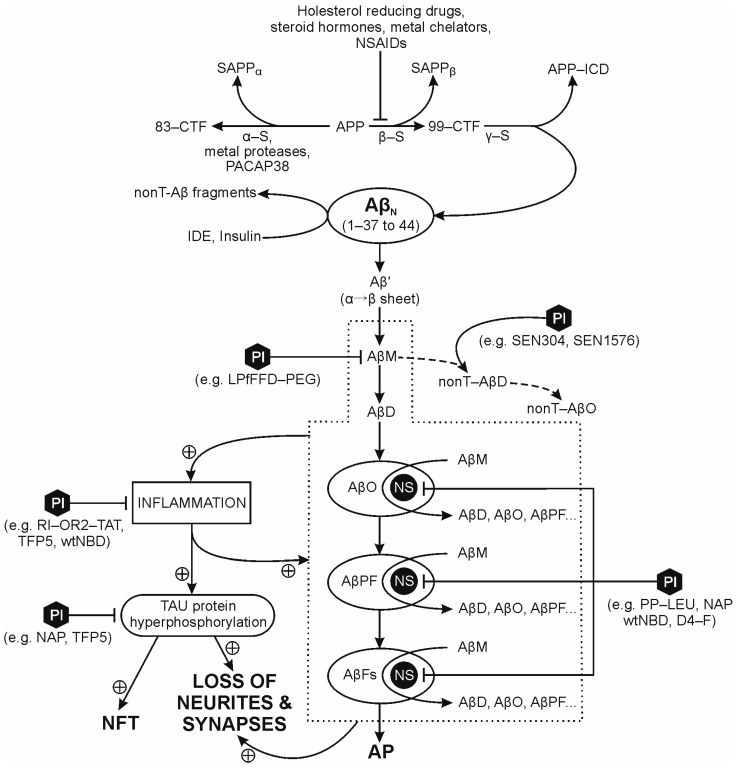
Pathways of amyloid precursor protein proteolysis, amyloid plaque formation and targets of selected peptide inhibitors. Abbreviations: 83-CTF (83-amino acid membrane bound *C*-terminal fragment); 99-CTF (99-amino acid membrane bound *C*-terminal fragment); AP (amyloid plaque); APP (amyloid precursor protein); APP-ICD (APP intracellular domain); Aβ′ (misfolded Aβ peptide with first α-sheet, then β-sheet structure); Aβn (native Aβ peptide with α-helix structure); AβD (amyloid β dimer); AβFs (amyloid β fibrils); AβM (amyloid β monomer); AβO (amyloid β oligomer); AβPF (amyloid β protofibril); IDE (insulin degrading enzyme); NFT (neurofibrillary tangles); nonT-AβD (non-toxic amyloid β dimer); nonT-AβO (non-toxic amyloid β oligomer); NS (nucleation site); NSAIDs (nonsteroidal anti-inflammatory drugs); PI (peptide inhibitor with examples in parentheses); SAPPα (soluble peptide APPα); SAPPβ (soluble peptide APPβ); ⊥ attenuates/inhibits, α-S (α-secretase); β-S (β-secretase); γ-S (γ-secretase); ⊕ promotes/accelerates.

The human amyloid precursor protein (APP) is a single-pass transmembrane protein in nerve cells. The physiological significance of APP is not fully understood, although it has a fast turnover rate in the mature, normal CNS [21,22]. APP is first processed in the endoplasmic reticulum and Golgi apparatus, and then transported either to synaptic terminals where it is inserted into the synaptic cell membrane [23] or into endosomes. The cell-membrane bound APP is processed by three competing pathways: (a) two amyloidogenic pathways; and (b) a non-amyloidogenic pathway (Figure 1). In the amyloidogenic pathways, APP is: (i) either sequentially cleaved by β- and γ-secretase leading to direct extracellular production of amyloid β-peptides (Aβps); or (ii) re-internalized into endosomes containing the β- and γ-secretases, leading to intracellular production of Aβps, that are either exported to the extracellular space (via vesicular transport) or degraded in lysosomes [21]. The APP re-internalization pathway substantially contributes to production of Aβps since: (a) β-secretase is located predominantly in endosomes [24]; and (b) 80% of Aβp release into the extracellular space is blocked by inhibiting endocytosis [23]. The amyloidogenic pathway first proteolyses APP with β-secretase into a soluble APPβ peptide and a 99-amino acid membrane bound *C*-terminal fragment (99-CTF)—and then proteolyses the 99-CTF with γ-secretase into a APP intracellular domain fragment (APP-ICD) and Aβps with 37–44 amino acid residues [21]. Low concentrations of Aβps are present in the central nervous system of non-demented individuals, therefore, Aβps could present a negative feedback loop in the regulation of synaptic plasticity and neuronal survival [25]. For example, the Aβ(1-42) monomer’s neuroprotective role is mediated by activation of the phosphatidylinositol-3-kinase pathway, and the stimulation of receptors of the insulin superfamily [26]. APP is cleaved by α- and then γ-secretase in the non-amyloidogenic pathway; γ-secretase activity is present on the cell surface and in endosomes [27,28] while α-secretase proteolysis of APP occurs mainly on the cell surface, and to a small extent in the Golgi apparatus [21]. APPα, the soluble product of α-secretase action on APP, has neuroprotective and memory-enhancing effects [29,30]. In physiological conditions, APP is preferentially processed by the non-amyloidogenic pathway and increased Aβp production correlates with decreased sAPPα levels [31,32,33,34]. Amyloid processing of APP is concentrated in lipid rafts, and non-amyloid processing in the non-raft regions of the cell membrane [35,36,37,38,39]. Proteolytic enzymes of both APP processing pathways are modulated by external factors. For example, some metalloproteases have an α secretase activity, contributing to the production of soluble sAPPα [40]. The amyloidogenic pathway activity is attenuated by cholesterol lowering drugs, metal chelators, steroid hormones or non-steroidal anti-inflammatory drugs [41]. Decreased cellular cholesterol levels disrupt the lipid raft’s structure and function, leading to a concomitant decrease in Aβp and an increase in sAPPα formation [42,43].

### 2.2. Pathological Processing of Soluble Amyloid β-Peptides (Aβps)

Aβ peptides are useful for neurons [26,44]. The key biochemical change in AD is the excessive accumulation of per-se non-toxic and soluble amyloid peptides (Aβ(1-37/44), in the intracellular and extracellular spaces, that alters proteostasis and triggers Aβ modification (e.g., by reactive oxygen species—ROS) into toxic intermediate Aβp products, misfolded soluble Aβps, Aβ dimers and Aβ oligomers. For example, intracellular *accumulation* of Aβ(1-42) per-se promotes increased ROS production and p53 mediated apoptosis [45,46,47,48]. The toxic intermediate Aβp products further aggregate into progressively less toxic and less soluble protofibrils, fibrils and finally extracellular brain senile plaques composed of proteinaceous deposit with β sheet structure. The most common Aβ is Aβ(1-40); Aβ(1-42) is the most susceptible to toxic conformational changes leading to nerve death and amyloid plaque formation. Aβ(1-42) monomers with intermediate conformations and Aβ(1-42) oligomers are the most neurotoxic and amyloid plaques the least [49,50]. Modified Aβ(1-42) peptides enter the cells via endocytosis and lead to lysosomal fusion dysfunction. The overall effect of lysosomal fusion dysfunction is an enhanced transport of vesicles by the exosomal pathway with increased shedding of modified Aβps into the extracellular space and a reduced Aβp digestion rate by macroautophagy [51]. Aβ(1-42) oligomers interfere with synaptic transmission by: (a) promoting neuronal death by attenuating NMDAR desensitization thus increasing the probability of intracellular Ca^2+^ overload [52,53]; (b) decreasing the density of AMPA synaptic receptors [54]; (c) uncoupling metabotropic glutamate receptors’ (mGluR5) dependent activation of PKC [55]; and (d) reducing glutamate reuptake thus promoting an increased NMDAR and mGluR5 mediated entry of Ca^2+^ [56]. AD studies on patients and animal models associated Aβ oligomers with synaptic dysfunction, cognitive decline, inhibition of hippocampal long-term potentiation (LTP) component in memory, and learning and memory impairment [57,58,59,60,61,62,63,64,65,66]. Aβ oligomers were better correlated with dementia and synaptic loss then Aβps in amyloid plaques [57,58].

### 2.3. Factors Promoting and/or Sustaining Pathological Processing of Amyloid β-Peptides (Aβps)

#### 2.3.1. Aβp Oxidation

Aβp oxidation promotes toxic misfolded Aβ monomers, oligomers and intermediate products [67]. For example, oxidation of Aβ(1-42) at the methionine residue 35, Aβ1-42-MET35-OX, promoted by H_2_O_2_ or copper ions, accelerates the production of toxic Aβ(1-42) products directly and indirectly by increasing oxidative stress, protein oxidation and lipid peroxidation [67,68]. In a cell model, ROS stabilize Aβ oligomers, by dityrosine cross-links in Aβ(1-42), and promote internalization of toxic Aβps into lysosomes [68]. Dityrosine crosslinked Aβ oligomers self-assemble to form amyloid fibrils; their presence was detected within plaques in brain samples of patients with AD [68].

#### 2.3.2. Mitochondrial (MITO) Dysfunction

Aβps and Aβ oligomers accumulate in MITO samples from transgenic mice overexpressing mutant AβPP and in post-mortem brains and from AD patients [69,70,71,72]. In human and animal studies, increased Aβp levels either preceded or followed MITO dysfunction implying a positive feedback loop. MITO dysfunction was due to: (a) oxidative modifications of key MITO enzymes (e.g., pyruvate dehydrogenase, isocitrate dehydrogenase, α-ketoglutarate dehydrogenase and cytochrome c oxidase) [70,72,73,74,75,76]; (b) reduced antioxidant defenses [77]; and (c) increased production of ROS [78]. Human and animal studies are in agreement that Aβp binds with MITO Aβ-binding alcohol dehydrogenase (ABAD) precipitating increased ROS generation, MITO dysfunction and cell death [79]. MITO dysfunction can also stimulate the amyloidogenic APP pathway; in a transgenic AD mouse model, knockout of manganese superoxide dismutase, a major MITO antioxidant enzyme, increased Aβp levels and amyloid plaque formation in the brain [77].

#### 2.3.3. Oxidative Stress

In AD, oxidative stress supports a self-sustained process of increased production of soluble Aβ oligomers from Aβs with a concomitant progressive failure of macroautophagy (reduced clearance of Aβs) and mitochondrial function (increased production of ROS). Oxidative stress upregulates β-secretase and γ-secretase expression thus promoting Aβ production [80,81,82,83,84,85]. Studies on human autopsy brain samples from patients with AD and on animal models of AD imply that oxidative damage occurs before Aβ plaque formation [86,87,88]. For example, an increase in reactive nitrogen species coincided with the onset of Aβ deposition in a transgenic AD mouse model [89].

#### 2.3.4. Advanced Glycation End Products (AGEs)

Patients with AD had more AGEs in brain samples than age-matched controls [90]; AGEs were co-localized with NFT and amyloid plaques [91], implying they accelerate aggregation of soluble Aβps and tau into amyloid plaques and NFTs respectively [90,92]. In a cell model, AGEs promoted oxidative stress and inflammation by activation of kappa-light-chain-enhancer of activated B cells (NF-κB) and increased cytokine IL6 gene expression with a concomitant increased release of Aβps [93].

#### 2.3.5. Apolipoprotein E (ApoE) Polymorphism and Cholesterol Levels

ApoE, the principal cholesterol carrier in the brain, is synthetized in astrocytes and transports cholesterol to neurons [94,95]. Persons with two APO-ε4 alleles have the single largest known genetic risk factor for late-onset sporadic AD [10,96,97,98] since APOE-ε4 does not promote the extra- and intra-cellular proteolysis of Aβps as efficiently as the APOE-ε2 or -ε3 isoforms [99]. This is consistent with the finding that MITO dysfunction in AD patients with ApoE-ε4 allele correlates better with cognitive dysfunction, than in AD patients carrying the ApoE-ε3 allele [100]. High serum total cholesterol is an independent risk factor for late onset AD in persons with any combination of ApoE alleles [13]. Supporting experimental evidence is provided by: (a) reduced Aβp production in cultured cells when cholesteryl-ester levels were reduced by inhibiting Acyl-CoA cholesterol acyltransferase (ACAT) [101]; (b) reduced amyloid and tau deposition, by enhancing autophagy with ACAT1 inhibition, in cell culture and whole animal mouse models of AD [102,103,104]; and (c) cholesterol depletion decreased Aβp production in rat hippocampal neurons [105].

#### 2.3.6. Inflammation, Vascular Pathology and Cellular Immunity

Human and animal model studies imply that inflammation promotes Aβ plaque deposition and tau hyperphosphorylation [22,106,107,108,109,110,111,112,113,114,115,116]. For example, post-mortem human AD brains have an increased activation of inflammatory and immune pathways with upregulated levels of pro-inflammatory cytokines, chemokines and complement proteins [108] consistent with microarray studies of brain samples from humans with AD or from animal models of AD that have an increased expression of genes involved in inflammation [109,111]. The intensity of inflammation varies over the course of AD; cytokine activity is the highest in the early clinical course of AD. The reduction of cytokine activity, in the late clinical course of AD, is concomitant with the increased brain levels of Aβ plaque, hyperphosphorylated tau, and AGEs [117]. Animal studies of AD transgenic mice imply that the intra-neuronal Aβ(1-42) is concentrated in neurites and synapses leading to their destruction and leaving a surviving nerve cell with a reduced number of neurites and synapses with adjacent extracellular accumulations of Aβ(1-42) [118]. The magnitude of brain synapse loss in AD correlates well with the degree of cognitive decline [119,120]. This could explain short term memory binding impairment in patients with AD, an early sign of the disease, since the patient’s capacity to form new memories is degraded by loss of synapses [121]. Chronic brain inflammation, as a result of extracellular Aβ(1-42) accumulation following destruction of synapses, neurites and finally neurons, is sustained by an inflammatory response of the surrounding astrocytes and microglia [122,123]. Inflammation increases ROS production that oxidize prostaglandins into F2α-isoprostanes. Both H_2_O_2_ and F2α-isoprostanes accelerate aggregation of Aβps into toxic oligomers [49,124]. Extracellular Aβ(1-42): (a) propagates its neurotoxic effects to neighboring nerve cells, an increased extracellular Aβ(1-42) leads to an increased intracellular Aβ(1-42) [18,125,126]; and (b) is further metabolized into less neurotoxic Aβ amyloid plaques by the concomitant inflammatory process [18,127,128,129]. Chronic inflammation, vascular pathology and cellular immune response in the brain are interdependent in AD and tend to re-inforce each other. Aβ(1-42) deposition in brain blood vessel walls accelerates its own brain deposition by promoting vascular damage with subsequent brain hypoperfusion, hypoxia and reduced Aβ(1-42) brain clearance. Thus, the observed vascular defects in AD can be either the cause or consequence of amyloid β accumulation [130]. The role of astroglia and microglia in the evolution of AD is under-investigated, although these cells are vital for normal nerve cell function [130]. Astroglia, a part of the tripartite synapse formed with the pre- and postsynaptic neurons, participates in synapse formation, pruning, maintenance and modulation of synaptic transmission; astroglia cells are also in contact with blood vessels and form, together with neurons, discrete neurovascular clusters [131,132]. Astroglia responds to Aβ(1-42) with an increased, NF-κB mediated, C3 release; C3 binds to neurons and promotes atrophy and elimination of synapses [133]. Astrogliosis, a reaction to brain damage, is present in patients with AD and can be reproduced in animal models of AD. Microglia cells also respond to Aβ(1-42) by promoting inflammation and Aβ(1-42) phagocytosis in the brain [130]. In addition to phagocytosis, microglia also regulate brain nerve cells’ function with synaptic pruning and apoptosis [134]. The role, or different roles over time, of astroglia and microglia on the progression of AD is not well understood; for a recent comprehensive review, see De Strooper and Karran, 2016 [130].

#### 2.3.7. Tau Processing in Alzheimer’s Disease

Tau phosphoprotein promotes tubulin assembly into microtubules and microtubule stability. In the brain, tau is concentrated in neurons and expressed at very low levels in astrocytes and oligodendrocytes [135]. In nerve cells, tau activity is not evenly distributed, but is concentrated at the distal end of axons, presumably to enable optimal stability and plasticity of synapses, the cell-to-cell connections between axon terminals of the upstream nerve and dendrites of the downstream nerve, that propagate the electrochemical stimulation. The breakdown of microtubules is promoted by tau phosphorylation with kinases or caspases; microtubule stability and growth are enhanced by phosphatases, promoting tau dephosphorylation [21,136,137]. Tau hyperphosphorylation leads to the disappearance of microtubules, the breakdown of intracellular traffic, the “dying back” of axons and tau redistribution from an axonal to a somato-dendritic pattern, presumably due to an increased tau synthesis as a response to increased tau hyperphosphorylation [138]. The degree of tau phosphorylation increases with age and is further accelerated by high blood glucose [139,140]. Additional factors that also promote tau hyperphosphorylation are: (a) Aβ(1-42) (via activation of kinases and caspases) [141,142,143]; (b) serine protease inhibitor α1-antichymotrypsin ACT [123]; and (c) oxidative stress [84,144,145,146]. Tau hyperphosphorylation promotes tau self-assembly and NFT formation [147].

### 2.4. Association of NFTs and Aβ Plaques with the Severity of Cognitive Decline in AD

Post-mortem studies on patients with AD imply a strong association among NFTs, neuronal loss and the severity of cognitive decline [148,149,150]. On the other hand, Aβ plaques seem to appear at the pre-symptomatic stage of AD and their levels stabilize relatively early in the disease process [151].

## 3. Peptides for Modifying Alzheimer’s Disease

Thus far, the efforts to develop AD therapies, based on “amyloid cascade hypothesis” and NFTs hypotheses, have been disappointing, even when the effective clearing of Aβ deposits in AD brain was demonstrated [152,153,154,155,156,157,158,159,160]. Alternative therapies with antioxidants [161] and anti-inflammatory agents [162,163] were also ineffective. Current drug therapy of AD is symptomatic, to support normal brain synaptic transmission with NMDAR antagonists (to attenuate excessive glutamate release and related nerve cell death rate) and with cholinesterase inhibitors, to conserve acetylcholine synaptic concentration [8,164].

The key issues for development of an efficient AD peptide therapy include: (a) selecting a target for peptide therapy; (b) understanding the relationships among protein sequence, structure, solubility and aggregation [165,166]; (c) integration of chemical kinetics into the drug discovery process to characterize and quantify the inhibition of protein aggregation [167]; (d) development of either Aβ peptide-sequence based or non-Aβ peptide sequence based peptide inhibitors of Aβ aggregation [168]; (e) selection of a drug delivery route [169]; and (f) development of a personalized therapy for AD patients [8,164,168].

### 3.1. Selecting a Target for Peptide Therapy

#### 3.1.1. Ameliorating AD Cognitive Impairment with Insulin

Brain regions with the highest concentration of insulin receptors are the olfactory bulb, hippocampus and hypothalamus [170,171]. The hippocampus, involved in long-term memory formation, spatial cognition, and conflict processing [172,173,174], has insulin receptors preferentially localized to nerve synapses [175,176]. Research on animal models implies that insulin receptor signaling contributes to long-term memory consolidation and improved spatial learning [171,177,178,179]. Insulin treatment improved memory and cognitive function in patients with AD and in patients with mild cognitive impairment [180,181,182,183,184]. The neuroprotective effects of insulin are mediated by several pathways. (a) Insulin binds to brain receptors and their activation: (i) increases transcription of anti-amyloidogenic proteins insulin-degrading enzyme and α-secretase; and (ii) decreases transcription of pro-amyloidogenic proteins AβPP, β-secretase and glycogen synthase kinase 3 α (Gsk3α) [185,186]. (b) Insulin attenuates Aβ oligomers’ binding to neurons thus protecting synapses against the toxic effects of Aβ oligomers [175]. (c) Insulin increases clearance of brain Aβ(1-42) into the CSF thus reducing Aβ(1-42)’s intracellular accumulation [187]. Insulin improved memory and cognition in patients with AD after a single dose and also after up-to four-months treatment [188,189,190,191,192]. The positive effects of insulin therapy diminish with the progression of AD when increased Aβp levels promote brain insulin resistance [193].

#### 3.1.2. Inhibition of Aβ Peptide Synthesis and Therapies with Tau Inhibitors, Aβ Peptide Chelators or Antibodies

Inhibitors were designed and tested that block APP expression or prevent proteolytic cleavage of APP into Aβps. Inhibition of γ-secretase, an enzyme involved in many physiological reactions, also lead to impaired lymphocyte differentiation and altered intestinal development [194,195]. β-secretase inhibitors are large molecules that are prevented by the blood–brain barrier (BBB) to reach their target [196,197]. Thus far, all inhibitors of β- or γ-secretase failed during clinical trials [198,199,200,201,202,203,204,205,206]. Chelation therapies, to disrupt interactions between Aβps and metal, were also not successful [207,208,209,210]. The only tau inhibitor molecule under clinical trials is phenothiazine methylene blue [211,212,213,214]. Antibody therapies for AD failed due to the antibodies’ inability to cross the BBB [215,216,217,218].

#### 3.1.3. Inhibition of Aβ Peptide Aggregation and Amyloid Formation

In principle, small molecules that inhibit Aβ aggregation [219,220,221,222,223,224] should not disrupt the normal biochemical processes in the body. However, their design is challenging for several reasons: (a) in contrast to protein-protein interactions that occur over large surface areas [225,226], protein-small molecule interactions occur over 3–5 times smaller contact surfaces [227,228]; and (b) regions of protein-protein interactions are relatively featureless and variable [229,230,231]. The discovery of key regions in the Aβ peptide sequence (i.e., *N*-terminus, hydrophobic core, hinge/turn region and *C*-terminus), responsible for Aβ plaque formation, was essential for the rational development of peptide inhibitors of amyloidogenesis [232,233,234,235,236,237,238].

Aβ peptides undergo several conformational changes: from an α-helix (i.e., random coil) native structure ⇒ to an intermediate α-sheet ⇒ to a β-sheet (i.e., highly ordered, with exposed hydrophobic amino acid residues that promote Aβp aggregation) structure present in amyloid fibrils, the building blocks of Aβ plaques. The intermediate α-sheet configuration has the propensity to aggregate into colloidal spheres that spontaneously form linear chains, the first step in amyloid fibril formation [239]. The conversion from the intermediate α-sheet to the β-sheet conformation has been inferred from protein crystal structure analysis [240,241,242] and studies with inhibitors of Aβ peptide aggregation [243]. Aβp intermediates and oligomers, that chronologically precede amyloid fibril formation, have heterogeneous conformations [244,245,246] that also change with age [247]. The Aβ plaques also contain a diverse population of truncated or modified Aβ peptides [248]. The Aβ peptide’s α-helix native structure can be stabilized or its β-sheet structure destabilized which has important implications for development of AD modifying therapies [196,249,250,251].

1. Aβp Sequence Derived Peptides

This group of peptides is based on the central hydrophobic core (CHC) sequence Aβ(16-20/22) and includes peptides with natural or modified amino-acids. The natural amino-acid peptides inhibit Aβ aggregation, but have the disadvantages of self-association into fibrils and low proteolytic stability. These disadvantages were ameliorated by various modifications including *N*-methylation [252], d-amino acid incorporation, retro-inverso peptides, replacement of amide by ester bonds, and cyclization [168].

• Natural Aβp sequence derived peptides

An example of a natural Aβ sequence derived peptide is the oligopeptide Ac-Leu-Pro-Phe-Phe-Asp-NH2 (iAb5p), where a proline and an aspartic acid amide substitute the valine and the alanine, respectively. This peptide crosses the BBB; in vivo, it inhibits formation of amyloid fibrils and promotes disassembly of formed amyloid plaques. The efficacy of this synthetic peptide is contributed to its proline residues that attenuate the β-sheet confirmation propensity of the natural Aβ peptides [249].

• Modified Aβp sequence derived peptides

Fluorinated Aβ sequence derived peptides are produced by fluorination of hydrophobic amino acids valine or phenylalanine. Fluorinated amino acids bind with hydrophobic residues of Aβps, thus preventing contact between Aβ peptide molecules and inhibiting their aggregation [253,254].

d-peptides are more resistant to proteolytic enzymes than their l-isomers. d-peptides inhibited Aβ aggregation in an animal model [255].

Retro-Inverso modified Aβ sequence derived peptides are derived from peptides by substituting the l-amino acids for their d-counterparts and reversing the sequence to mimic the original peptide since they retain the same spatial positioning of the side chains and 3D structure [244,256,257]. These peptides combine the advantage of the original peptide (i.e., inhibition of aggregation) with an increased resistance to enzymatic proteolysis, a reduced self-aggregation and an improved ability to cross the BBB in an animal model [258,259,260,261,262].

Cyclic peptides were developed from KLVFF derivatives. They are strong and specific inhibitors of amyloid formation. Compared to their noncyclic equivalents, they are metabolized more slowly due to their resistance to enzymatic degradation [244,263,264].

*N*-methylated peptides (e.g., SEN304 and SEN1576) inhibit Aβp mediated toxicity in an animal model. They do not inhibit Aβp aggregation, they divert Aβp aggregation into non-toxic forms and remove toxic oligomers [265,266].

α sheet peptides have alternating d-amino acids and l-amino acids; they reduce Aβ aggregation and toxicity but lack the ability to cross the BBB [267].

2. Peptides not derived from Aβp sequence

Peptides not derived from Aβp sequence and peptides derived from Aβ sequence share the qualities of hydrophobicity and the ability to incorporate into the β-sheets structure, thus inhibiting Aβp aggregation.

• Peptides not derived from Aβp sequence containing natural amino acids

Examples are NAP [268], carnosine [269,270] and hexapeptides [271]. All of them inhibit Aβ peptide aggregation. NAP also attenuated mild cognitive impairment in phase II clinical trials [272,273].

• Peptides not derived from Aβp sequence containing modified amino acids

D-4F improved cognitive function when administered orally to mice and also inhibited Aβp deposition [274].

Cyclic peptide PP-Leu is a tridecapeptide analogue of theta-defensins. This peptide combines the ability to inhibit Aβ oligomer and amyloid fibril formation, by sequestration of amyloidogenic Aβ peptides into colloid-like assemblies, with a high resistance to endoproteinase K [275].

### 3.2. Relationship between Protein Sequence, Structure, Solubility and Aggregation

Protein aggregation is regulated by the same physicochemical laws as normal protein folding and shares many common features with other thermodynamically stable protein conformations [276]. Protein solubility is an essential feature for successful development of AD peptide therapy; proteins in aggregates lose their therapeutic effect, can become toxic or increase the probability for an immune response in the treated patient [277,278].

Protein aggregation prediction methods are based on the analysis of physicochemical factors that determine protein thermodynamics and kinetics of unfolded polypeptide aggregation [279,280], the discovery of key regions in the Aβ peptide sequence responsible for Aβ plaque formation [281,282,283,284,285,286,287,288] and advances in modelling and simulation of aggregation-prone regions, recently reviewed by Trainor et al. [166]. The discovery of aggregation-prone regions in Aβ peptides is essential for the rational development of peptides that reduce the Aβ peptides’ tendency to form ordered intermolecular assemblies [289,290,291]. The regions of Aβ(1-42) and Aβ(1-40), with a high aggregation propensity, are the central region (residues 18–22) and the *C*-terminal region (residues 32–42) [236,292,293]. In solution, Aβ peptides are largely unstructured with no stable folded structure [165].

Aggregation prediction algorithms were classified by Trainor et al. into amino acid composition-based algorithms, sliding window/pattern-based algorithms and tertiary/quaternary structure-based algorithms [166].

#### 3.2.1. Amino Acid Composition-Based Algorithms

Amino acid composition-based algorithms use statistical analyses and machine learning algorithms to predict/generalize aggregation-prone regions based on amino acid sequences of proteins with known aggregation propensities. These algorithms use amino acid and/or dipeptide frequencies to determine peptide solubility in a binary manner (either soluble or insoluble) and disregard the position of each amino acid in the studied peptide sequence. Therefore, they cannot pinpoint specific amino acid changes with the greatest aggregation impact [3,166,279,294,295,296,297,298,299]. To improve solubility predictions of amino acid composition-based algorithms, parameters are included that reflect the order of the amino acids in the studied protein sequence, for example Shannon entropy and parameters derived from Chaos Game Representations of amino acid sequences [296,300]. To summarize, these methods are more appropriate for binary, proteome wide, solubility surveys and less for optimizing protein solubility of AD modifying peptides [166].

#### 3.2.2. Sliding Window/Pattern-Based Algorithms

The sliding window methods can analyze amino acid sequences for aggregation-prone regions that can be as short as five amino acid residues. They are based on the theoretically and experimentally verified premise that 5–7 residue sequence segments can exert a disproportionate influence on protein solubility. Protein solubility is estimated by considering the contributions of charge, hydrophobicity/hydrophilicity ratio, secondary structure propensity, statistical analysis of residue pairings between adjacent β-strands in known structures and patterns of residue distribution in amyloidogenic hexapeptides [165,282,301,302,303,304,305,306,307,308,309,310,311,312,313,314,315,316,317]. CamSOL incorporates the sliding window method to develop soluble antibodies targeting Aβ peptides [313]. Other examples of tools using the sliding window method to study Aβ peptides aggregation are AmyloidMutants [310], STITCHER [318], AGGRESCAN [301,316] and Ziggregator [165]. The sliding window based methods can recognize amino acid sequence features relevant to aggregation of Aβ peptides. However, the composition of Aβ peptide aggregates varies, is not homogenous [287]. In addition, most sliding window method based tools do not include information on protein structure that influences exposure of aggregation prone regions; a limited exposure of aggregation prone regions can change the agreement between aggregation propensity predictions and experimentally measured aggregation [166]. None of the sliding window methods explicitly take into account the tertiary/quaternary structure of a native protein although associations between natively folded proteins can indirectly impact aggregation kinetics of partially or fully unfolded proteins [277]; in addition, aggregates may be formed by partially unfolded/structured proteins [319,320,321].

#### 3.2.3. Tertiary/Quaternary Structure-Based Algorithms

Structure based methods [313,322,323,324,325] reliably predict protein aggregation even when a significant number of aggregation-prone regions is inaccessible; for example when transient (α-sheet) or stable (β-sheet) associations between folded Aβ proteins accelerate aggregation [166]. Examples of structure based methods are SAP [326], the upgraded version of CamSol [165] and AGGRESCAN3D [166]. The SAP method can grade cytotoxicity of Aβ(1-42) peptide variants [326].

### 3.3. Integration of Chemical Kinetics into the Drug Discovery Process to Charaterize and Quantify the Inhibition of Protein Aggregation

#### 3.3.1. Challenges in Understanding Aggregation Inhibition Mechanisms at the Molecular Level

The key step in Aβ plaque formation are conformational changes of Aβ peptides into reactive, metastable oligomers (i.e., the primary nucleation processes) that assemble into protofibrils and later fibrils [327]. Aggregation of Aβ peptides is further accelerated by the secondary nucleation processes triggered by fibril fragmentation and fibril surface catalyzed nucleation [328,329]. The kinetics of fibril formation follow a sigmoidal curve, preceded by a lag phase and terminated by a plateau after Aβ monomer depletion [330,331]. Most inhibitors attenuate Aβ peptide aggregation by forming covalent or noncovalent bonds with one or more products of the aggregation pathway. For example, aromatic compounds sequester protein monomers and/or oligomers in a nonspecific manner [332,333]. A variety of methods is necessary to quantify the binding characteristics of Aβ peptides’ monomers and oligomers with peptide inhibitors [50,333,334,335,336,337,338,339].

#### 3.3.2. Kinetic Analysis of Aggregation Inhibition Mechanisms Is Necessary for Development and Evaluation of Drug-Like Small Molecules

Arosio et al. present a strategy that integrates experimental characterization of Aβ peptide products with chemical kinetics (i.e., reaction kinetics) to facilitate a rational design and evaluation of peptides for AD therapy [167]. The advantage of chemical kinetics is the method’s ability to measure very weak binding events and their effects, an important characteristic of inhibitor–protein interactions. This method evaluates how different experimental conditions influence the speed of reactions that generate Aβ oligomers, protofibrils and fibrils, yielding information about the reactions’ mechanisms and transition states [340,341]. Chemical kinetics can model the effect of: (a) protein structural changes due to binding [342]; (b) covalent reactions between proteins and inhibitors [343]; (c) off-pathways (i.e., non-amyloidogenic pathways) that also produce toxic soluble oligomers [50]; and (d) Aβ aggregation inhibitors that reduce the amount of toxic Aβ oligomers by redirecting the aggregation process to alternative off-pathway products [334,339,343,344,345].

Kinetic analysis of Aβ peptide aggregation inhibition mechanisms evaluates how the inhibitions of primary nucleation, secondary nucleation or elongation individually contribute to Aβ fibril formation. For example: (a) inhibition of monomer formation decreases the rate of primary and secondary nucleation and fibril elongation; (b) inhibition of oligomer production reduces primary and secondary nucleation rates; (c) inhibitor binding to the fibril ends slows down the fibril elongation process; and (d) inhibitor binding to amyloid fibrils decreases only the surface catalyzed secondary nucleation rate [167]. In addition, kinetic analysis can: (a) distinguish between the effects of fibril elongation and secondary nucleation on lag-time phases and growth rates of fibril mass fraction; (b) quantify the effect of primary nucleation inhibition on the lag-time preceding the fibril mass growth phase; (c) demonstrate that the final quantity of fibril mass fraction is reduced only by the inhibition of primary nucleation and fibril elongation processes; and (d) demonstrate that the inhibition of primary nucleation and fibril elongation processes delay formation of the toxic Aβ oligomer intermediates, but only the inhibition of secondary nucleation attenuates the maximum amount of toxic Aβ oligomers [167].

In summary, the chemical kinetics data can be used to build mathematical models of Aβp forming reactions to: (a) better understand and describe chemical processes governing peptide inhibitor-Aβ peptide products interactions; and (b) to design or modify peptide inhibitors of Aβ peptide aggregation [330,331,337,346,347,348].

### 3.4. Selection of Drug Delivery Route

Efficient brain delivery over the BBB is essential for peptide based AD treatment [169]. Intravenous peptide administration, in an animal model, leads to a brain peptide content of less <1% of the administered dose [349]. Therefore, intraperitoneal injection or intracerebral infusion are the preferred drug delivery routes to study the effect of anti-AD peptides in animal models [350,351,352,353,354]. For example, intraperitoneal injection of TFP5, an inhibitor Cdk5/p25 activity, attenuated tau hyperphosphorylation, neurofilament formation, Aβp accumulation and inflammation in mice brains [355]. In human, intraperitoneal injection or intracerebral infusion are not practical administration routes for long term treatment. An alternative to both, oral administration, was tested in animals with the anti-AD peptide D3; however, the peptide only elicited an improvement in cognitive behavior and a reduction in amyloid deposition with the use of very large doses (0.5–1 mg/mouse/day) [356]. Experiments with radioactively labeled exendin (9–39) imply that intranasal delivery is the only non-invasive drug delivery route, studied in an animal model, that could be used as a testing model for long term treatment in human; it has also a considerably higher peptide delivery to the brain than intravenous administration [357]. The use of polyethylenimine (PEI) conjugated peptides (e.g., V24P(10-40)-PEI), or peptides with a cell-penetrating peptide segment with multiple positively charged residues (e.g., wtNBD), further increase peptide brain delivery efficiency, to more than 17% of the intranasally administered dose [358,359].

Intranasal application of potential anti-AD peptides for human treatment (e.g., wtNBD, PACAP38 and NAP) reduced amyloidopathy in animal models by targeting diverse amyloidogenic pathways. For example: (a) wtNBD inhibits the induction of NF-κB activation, suppresses microglial activation, attenuates Aβ plaque deposition and improves cognition [358]; (b) PACAP38 increases α-secretase activity and improves cognition [360]; and (c) NAP promotes microtubule assembly, attenuates Aβ peptide accumulation and tau hyperphosphorylation [273,361]. The efficiency of intranasally delivered antiamyloidogenic peptides could be further enhanced by increasing their protease resistance [169]. The strategy to treat AD in human, with intranasal application of peptides, is also vindicated by phase 2 clinical trials on AD patients with intranasal insulin application that improved cognition [181,183,189,191,362,363,364,365].

### 3.5. Summary of Presented AD Modifying Peptides

A summary of AD modifying peptides, including references, is presented in Table 1.

### 3.6. Development of a Personalized Therapy for AD Patients

Several research groups have recently articulated the need for a personalized approach to the treatment of patients with AD, a multifactorial disease due to a combination of genes and environmental factors [8,164,169]. Peng et al. proposed a stage-specific strategy for a comprehensive and personalized treatment of patients with AD. This strategy includes genome editing of AD associated mutations, physical activity, brain stimulation, adequate social communication, appropriate diet, multiple drug therapy targeting amyloidogenesis and inflammation or stimulating brain blood flow and neuronal regeneration with stem cell technology [164].

## 4. Conclusions

AD is a progressive neurodegenerative disorder, where the rate of disease progression varies considerably from person to person due to life style, genetic and environmental factors. Chronic inflammation, vascular pathology and accumulation of toxic Aβ peptide products tend to sustain and re-inforce each other overtime, and these processes are accompanied by changes in brain structure, connectivity and function. Therefore, an effective peptide based AD therapy has to be personalized and stage specific. Chemical kinetics and aggregation prediction algorithms are essential tools for the development of peptide based AD modifying drugs, and intranasal application is the preferred delivery route for the development and treatment with anti-amyloidogenic peptides.

## Figures and Tables

**Table 1:** AD modifying peptides with brief description. Abbreviations: act. (activity); agg. (aggregation); att. (attenuates); hyperph. (hyperphosphorylation); infl. (inflammation); NFF (neurofilament formation); pII. c.t. (phase II clinical trials); pl. form. (plaque formation); ref. (reference); stim. (stimulates); α-S (α-secretase); ● (observed effect).

Peptide Name and Ref.	Att. Brain Infl.	Att. Aβp Agg./Pl. Form.	Att. Tau Hyperph./NFF	Stim. Neurog.	Improves Cognition	Stim. α-S Act.
Ac-Leu-Pro-Phe-Phe-Asp-NH2 (iAb5p), [249]		●				
LPfFFD-PEG, [253,254]		●				
D-(PGKLVYA), [255]		●				
RI-OR2-TAT, [260]	●	●		●		
cyclo(17, 21)-(Lys17, Asp21)Aβ(1-28), [366]		●				
SEN304, SEN1576, [265,266]		●				
α sheet peptides, [267]		●				
NAP, [268,273,361]		●	●		● (pII. c.t.)	
D4-F, [274]		●			●	
D3, [356]		●			●	
PP-Leu, [275]		●				
TFP5, [355]	●	●	●			
wtNBD, [358]	●	●			●	
PACAP38, [360]					●	●
insulin, [181,183,189,191,362,363,364,365]					● (pII. c.t.)	
